# Antisense oligonucleotide is a promising intervention for liver diseases

**DOI:** 10.3389/fphar.2022.1061842

**Published:** 2022-12-09

**Authors:** Kailing Lu, Qijing Fan, Xiaoju Zou

**Affiliations:** ^1^ College of Chinese Materia Medica and Yunnan Key Laboratory of Southern Medicinal Utilization, Yunnan University of Chinese Medicine, Kunming, Yunnan, China; ^2^ Center for Life Sciences, School of Life Sciences, Yunnan University, Kunming, Yunnan, China

**Keywords:** antisense oligonucleotide (ASO), NAFLD, hepatitis, liver fibrosis, liver cancer

## Abstract

As the body’s critical metabolic organ, the liver plays an essential role in maintaining proper body homeostasis. However, as people’s living standards have improved and the number of unhealthy lifestyles has increased, the liver has become overburdened. These have made liver disease one of the leading causes of death worldwide. Under the influence of adverse factors, liver disease progresses from simple steatosis to hepatitis, to liver fibrosis, and finally to cirrhosis and cancer, followed by increased mortality. Until now, there has been a lack of accepted effective treatments for liver disease. Based on current research, antisense oligonucleotide (ASO), as an alternative intervention for liver diseases, is expected to be an effective treatment due to its high efficiency, low toxicity, low dosage, strong specificity, and additional positive characteristics. In this review, we will first introduce the design, modification, delivery, and the mechanisms of ASO, and then summarize the application of ASO in liver disease treatment, including in non-alcoholic fatty liver disease (NAFLD), hepatitis, liver fibrosis, and liver cancer. Finally, we discuss challenges and perspectives on the transfer of ASO drugs into clinical use. This review provides a current and comprehensive understanding of the integrative and systematic functions of ASO for its use in liver disease.

## 1 Introduction

The liver is the largest “chemical factory” in the human body. It plays a crucial role in nutrient metabolism, energy generation, detoxification, hormone inactivation, bile production, regulation of blood coagulation, immune defense, and many other processes. However, with the increasing availability of processed foods and the spread of unhealthy lifestyles, the liver can become overburdened. Liver-related diseases are becoming a global epidemic and are one of the leading causes of death worldwide. According to statistics, in 2017, there were 2.14 million liver-related deaths, representing an 11.4% increase since 2012 (Paik et al., 2020).

Numerous predisposing factors contribute to the development of liver disease, such as viral infections, alcohol use, autoimmune diseases, cholestasis, drug use, and metabolic diseases. Liver diseases follow a progression of severity, from hepatic steatosis, to hepatitis, liver fibrosis, and eventually culminating in liver cirrhosis and liver cancer (Anstee et al., 2019). Currently, major treatments for liver-related diseases include lifestyle interventions, drug therapy, and liver transplantation. Of the three, drug therapy has been the dominant approach, compared to the limitations of lifestyle interventions and liver transplantation. Drugs, including small molecule compounds, antibody drugs, polymeric DNA and small interfering RNA nucleic acid drugs, etc., administered alone or in combination, have been approved by the U.S. Food and Drug Administration (FDA) for the treatment of primary biliary cholangitis (PBC), acute hepatic porphyria, parenteral nutrition-associated cholestasis, hepatic veno-occlusive disease, hepatitis C (HCV), hepatitis B (HBV), and liver cancer. However, thus far there is no FDA- approved drug treatment for fatty liver, non-alcoholic steatohepatitis (NASH), and liver fibrosis. Therefore, new drug discovery and development for liver-related diseases is extremely urgent due to unmet clinical needs.

In 1978, Stephenson and Zamecnik (1978) first used Antisense oligonucleotide (ASO) for therapeutic purposes, and subsequently developed multiple ASO for the treatment of diseases. In a landmark move in 1998, the FDA approved Fomivirsen, the first ASO drug, for the treatment of cytomegalovirus (CMV) retinitis. Over the last two decades, the use of ASO drugs has become an increasingly important therapeutic strategy. Prior to 2009, only two ASO drugs were approved. From 2010 to 2021, the FDA approved a total of eight ASO drugs (Table 1) (Hair et al., 2013; Syed, 2016; Hoy, 2017; Keam, 2018; Dhillon, 2020; Heo, 2020; Brown and Wobst, 2021; Shirley, 2021). Among these, Defibrotide, the first ASO drug approved for the treatment of liver-related disease (Choy, 2016), has been used for the treatment of adult or pediatric patients with hepatic veno-occlusive disease (VOD). In recent years, research on ASO therapy for liver disease has been active as never before. Improved ASO design and optimized synthetic nucleic acid chemistry, combined with the development of highly selective and efficient conjugate delivery technology platforms, have established and validated ASO as a new class of drugs.

**TABLE 1:** Features and properties of FDA-approved ASO drugs.

Year of approval	Drugs	Disease	Targets	Chemical modifications	Mechanism	Backbone (size)	Route of administration
1998	Fomivirsen	CMV retinitis	CMV IE2	Phosphorothioate	Activate the RNase H	GCG​TTT​GCT​CTT​CTT​CTT​GCG (21)	Intravitreal
2004	Pegaptanib	AMD	VEGF receptor	Purine ribose sugars are 2′-O-methylated and pyrimidine ribose sugars all 2′-fluorinated	Pegylated aptamer by targeting VEGF receptor	CGG​AAU​CAG​UGA​AUG​CUU​AUA​CAU​UCG (27)	Intravitreal
2013	Mipomersen	HoFH	Apo B	Phosphorothioate, 2′-MOE	Activate the RNase H	GCC​UCA​GTC​TGC​TTC​GCA​CC (20)	Subcutaneous
2016	Eteplirsen	DMD	Dystrophin exon 51	PMO	Regulate the splicing of RNA	CTC​CAA​CAT​CAA​GGA​AGA​TGG​CAT​TTC​TAG (30)	Intravenous
2016	Nusinersen	SMA	Survival motor neuron 2 (SMN2)	Phosphorothioate, 2′-MOE	Regulate the splicing of RNA	UCACUUUCAUAAUGCUGG (18)	Intrathecal
2016	Defibrotide	VOD	Multiple targets	A natural product	Not fully elucidated	A polydisperse mixture	Intravenous
2018	Inotersen	hATTR	Transthyretin (TTR)	Phosphorothioate, 2′-MOE	Activate the RNase H	UCU​UGG​TTA​CAT​GAA​AUC​CC (20)	Subcutaneous
2019	Golodirsen	DMD	Dystrophin exon 53	PMO	Regulate the splicing of RNA	GTT​GCC​TCC​GGT​TCT​GAA​GGT​GTT​C (25)	Intravenous
2020	Viltolarsen	DMD	Dystrophin exon 53	PMO	Regulate the splicing of RNA	CCT​CCG​GTT​CTG​AAG​GTT​TC (20)	Intravenous
2021	Casimersen	DMD	Dystrophin exon 45	PMO	Regulate the splicing of RNA	CAA​TGC​CAT​CCT​GGA​GTT​CCT​G (22)	Intravenous

## 2 Antisense oligonucleotide

ASO typically consists of 10–30 single-stranded DNA nucleotides (Opalinska and Gewirtz, 2002), that are complementary to the target mRNA. Through the principle of Watson-Crick base pairing, ASO can accurately target the transcription product of this specific gene, thereby exhibiting a precise function in inhibiting gene expression (Crooke et al., 2021). Due to its strong specificity, high efficiency, low toxicity and low dosage, ASO could potentially serve as a new therapeutic direction for drug development in liver diseases. It is important to note that ASO drugs have different absorption and metabolism pathways than traditional small molecule drugs due to their unique properties. ASO drugs are mainly administered by intravenous and subcutaneous routes (Tables 1, 2). When they enter the body, they are mainly degraded by endonucleases and exonucleases in the blood and target organs. In addition, they are not metabolized by the liver and hepatic microsomes, as they are not substrates of the hepatic drug enzyme P450s. When ASOs are metabolized to short oligonucleotides of various sizes, they are mainly excreted into the urethra by membrane leakage, vesicle release, or exosome release (Migliorati et al., 2022). It can be seen that pharmacokinetic parameters such as absorption, distribution, metabolism and excretion of ASO drugs have an essential influence on the dose and toxicity of ASO drugs, and future studies should pay more attention to this aspect.

**TABLE 2:** A summary of studies on the application of ASOs in liver diseases.

Mechanism	Targets	Chemical modifications	Backbone (size)	Route of administration
**NAFLD**				
Inhibits lipid synthesis	*Pnpla3*	2′-4′ constrained ethyl (cEt)	TATTTTTGGTGTATCC (16)	Subcutaneous
	*Srebp-1c*	Phosphorothioate	GTT​CAA​GAG​CCG​CCT​CAC​CG (20)	Intraperitoneal
	*Dgat2*	phosphorothioate, 2′-MOE	GCA​TTA​CCA​CTC​CCA​TTC​TT (20)	Intraperitoneal
	*Psd3*	Phosphorothioate, 2′-O-ethyl	GTATTAATACTCTCTC (16) CTTGATCGAGAATCCT (16)	Subcutaneous
	*Adrp(Plin2)*	Phosphorothioate, 2′-MOE	GGT​CAT​CTG​GCC​AGC​AAC​AT (20)	Intraperitoneal
	*Tip47(Plin3)*	Phosphorothioate, 2′-MOE	CAC​AGT​GTT​GTC​TAG​GGC​CT (20)	Intraperitoneal
	*Mst3*	Phosphorothioate, 2′-4′ constrained ethyl	ACGCTATATACAATCT (16) GTTAATTTTAGGTCTC (16)	Intraperitoneal
	*Stk25*	Phosphorothioate, 2′-4′ constrained ethyl	GCATAATCCCCTAGGC (16)	Subcutaneous
	*Creb*	Phosphorothioate, 2′-MOE		Intraperitoneal
	*Ncst*			Intraperitoneal
	*miRNA-21*	LNA		Intravenous
Promotes lipid oxidation	*Fgfr4*		GCC​ACA​TTT​CCT​TCC​AGC​TG (20) TCC​ATT​TCC​TCA​GAG​GCC​TC (20)	Subcutaneous
	*Scd1*		GTG​TTT​CTG​AGA​ACT​TGT​GG (20)	Intraperitoneal
	*Acc1*	Phosphorothioate, 2′-MOE	CGT​GGG​ATG​CCT​TCT​GCT​CT (20)	Intraperitoneal
	*Acc2*	Phosphorothioate, 2′-MOE	GAG​TTC​CTC​TGC​TGA​CTG​GC (20)	Intraperitoneal
	*Hsd11β1*		TGT​TGC​AAG​AAT​TTC​TCA​TG (20)	Intraperitoneal
	*(p)rr*	Phosphorothioate, 5′-methyl, 2′-4′ constrained ethyl	AGATATTGGTCCATTT (16)	Subcutaneous
	*ApoB*	Phosphorothioate, 2′-MOE	GCC​UCA​GTC​TGC​TTC​GCA​CC (20)	Intraperitoneal
Others	*Mat1α*		CCA​CTT​GTC​ATC​ACT​CTG​GT (20)	Intraperitoneal
	*Fabp3*	Morpholino	TGC​CGA​TAA​AAG​CGT​CTG​CCA​TGT​T (25)	Intraperitoneal
	*Angptl8*	2′-MOE		Intraperitoneal
**Hepatisis**				
Inhibits HC apoptosis	*Tnf-α*	Phosphorothioate	TGA​TCC​ACT​CCC​CCC​TCC​ACT (21)	Intravenous
	*bid*	Phosphorothioate, 2′-MOE	GAC​CAT​GTC​CTG​GCC​AGA​AA (20)	Intraperitoneal
	*Fas*	Phosphorothioate, 2′-MOE	TCC​AGC​ACT​TTC​TTT​TCC​GG (20)	Intraperitoneal
Inhibits inflammatory infiltration	*Sab*	2′-MOE	GCT​GCC​GCT​ACA​GGG​AAT​GC (20)	Intraperitoneal
	*Stk25*	Phosphorothioate, 2′-4′ constrained ethyl	GCATAATCCCCTAGGC (16)	Subcutaneous
	*Psd3*	Phosphorothioate, 2′-O-ethyl	GTATTAATACTCTCTC (16) CTTGATCGAGAATCCT (16)	Subcutaneous
**Liver fibrosis**				
Promotes ECM degradation	*Timp-1*	Phosphorothioate	GGC​GCC​ATC​GTG​GTA​TCT​GC (20) GCT​CTA​GCG​TGT​CTC​TAG​GA (20) GAT​AAA​CAG​TGT​TCA​GGC​TTC (21) GTT​CAG​GCT​TCA​GCT​TTT​GC (20)	Intravenous
	*Timp-2*	Phosphorothioate	GCA​AAC​GTT​ACG​TCT​ACA​TC (20) GAT​CAG​CTC​TTG​AGG​ACG​AA (20)	Subcutaneous
Inhibits collagen synthesis	*Hsp47*	Phosphorothioate	GAA​GCA​GGA​GGG​AGC​GCA​TG (20)	Cell level
	*Sab*	2′-MOE	GCT​GCC​GCT​ACA​GGG​AAT​GC (20)	Intraperitoneal
	*Periostin*		CAC​CAC​TGT​TCG​TAA​UUU​GG (20)	Intraperitoneal
	*Jag1*	Phosphorothioate, 2′-O-methyl, 2′-fluoro	GCGATACTGAGATGGC (16)	Intraperitoneal
	*Il-4rα*	Phosphorothioate, 2′-4′ constrained ethyl, 2′-MOE	CCG​CTG​TTC​TCA​GGT​GAC​AT (20)	Intraperitoneal
Inhibits collagen synthesis	*Tgf-β*	Phosphorothioate, LNA	CAAAGTATTTGGTCTCC (17)	Subcutaneous
Inhibits HSC activation	*Stat3*	Phosphorothioate, 2′-MOE	CTATTTGGATGTCAGC (16)	Intraperitoneal
Promotes HSC apoptosis	*Bcl-x*	Phosphorothioate, 2′-O-ME	TGG​TTC​TTA​CCC​AGC​CGC​CG (20)	Cell level
Promotes RE recover	*Dgat1*			Intraperitoneal
**Liver cancer**				
Promotes tumor cell apoptosis	*Mcl-1*	Phosphorothioate, 2′-MOE	TTG​GCT​TTG​TGT​CCT​TGG​CG (20)	Cell level
	*Ae-2*		GCG​CTG​CTC​ATG​GCC​GAA​TCT (21)	Cell level
	*Myc*	Phosphorothioate	AACGTTGAGGGGCAT (15)	Intravenous
	*Survivin*	Phosphorothioate, 2′-MOE	TGTGCTATTCTGTGAATT (18)	Intravenous
	*Pkc-α*		CAG​CCA​TGG​TTC​CCC​CCA​AC (20)	Cell level
	*Mk*		CCC​CGG​GCC​GCC​CTT​CTT​CA (20)	Cell level
	*Hif-1α*		ACG​CAG​AAT​ATA​TTC​CAT​GA (20)	
Inhibits tumor cell proliferation, migration, invasion	*Elk-1*		CAG​CGT​CAC​AGA​TGG​GTC​CAT (21)	Subcutaneous
	*c-raf.1*	Phosphorothioate	UCC​CGC​CUG​UGA​CAU​CGA​UU (20)	Intravenous
	*miR-96*		GCA​AAA​AUG​UGC​UAG​UGC​CAA​A (22)	Cell level
	*Hif-1α*		ACG​CAG​AAT​ATA​TTC​CAT​GA (20)	
	*Myc*	Phosphorothioate	AACGTTGAGGGGCAT (15)	Intravenous
	*Arl4c*	Phosphorothioate, LNA	GCATACCTCAGGTAA (15) GAA​GGT​TAC​AGT​ATT​TGG​C (19)	Subcutaneous
	*Hlnc1*		GUA​CAT​AGG​CGT​ACA​UGA​UU (20)	Intraperitoneal
	*miR-221*	Phosphorothioate, 2′-O-ME	GAA​ACC​CAG​CAG​ACA​AUG​UAG​CU (23)	Cell level
Inhibits immune evasion	*Hnrnpm*	LNA	TCG​ATA​CGA​GAC​CTC​TGA​ATT​TTC​T (25) TCG​CCG​ACA​TCA​AGA​TGG​AGA​ATC​T (25) TCC​ATG​GAG​CGC​ATT​GGC​TCT​GTC​T (25)	Intraperitoneal
	*Mtdh*	LNA	ACGCATCTGAGTACTTG (17) GCTGGGTCACTAGTGCT (17)	Intraperitoneal
	*Myc*	Phosphorothioate	AACGTTGAGGGGCAT (15)	Intravenous
Inhibits metastasis	*Stat3*	Phosphorothioate, 2′-MOE	GCT​CCA​GCA​TCT​GCT​GCT​TC (20)	Intraperitoneal
	*Arl4c*	Phosphorothioate, LNA	GCATACCTCAGGTAA (15) GAA​GGT​TAC​AGT​ATT​TGG​C (19)	Subcutaneous
	*Cd10*	Phosphorothioate	TTC​TGA​CTT​GCC​CAT​CAC​CT (20)	Intraperitoneal
	*Opn*	Phosphorothioate, 2′-MOE	ACT​TCG​GTT​GCT​GGC​AGG​TC (20)	Intraperitoneal
Inhibits HC apoptosis	*Tnfr1*	2′-MOE		Intraperitoneal
Inhibits telomerase activity	*h-Tert*	Phosphorothioate	ACT​CAC​TCA​GGC​CTC​AGA​CT (20)	Intravenous
Inhibits de-differentiation	*Smyd3*	2′-4′constrained ethyl	CGTATTAACACTGGCA (16)	Subcutaneous

### 2.1 The mechanism of action of antisense oligonucleotide

**FIGURE 1:**
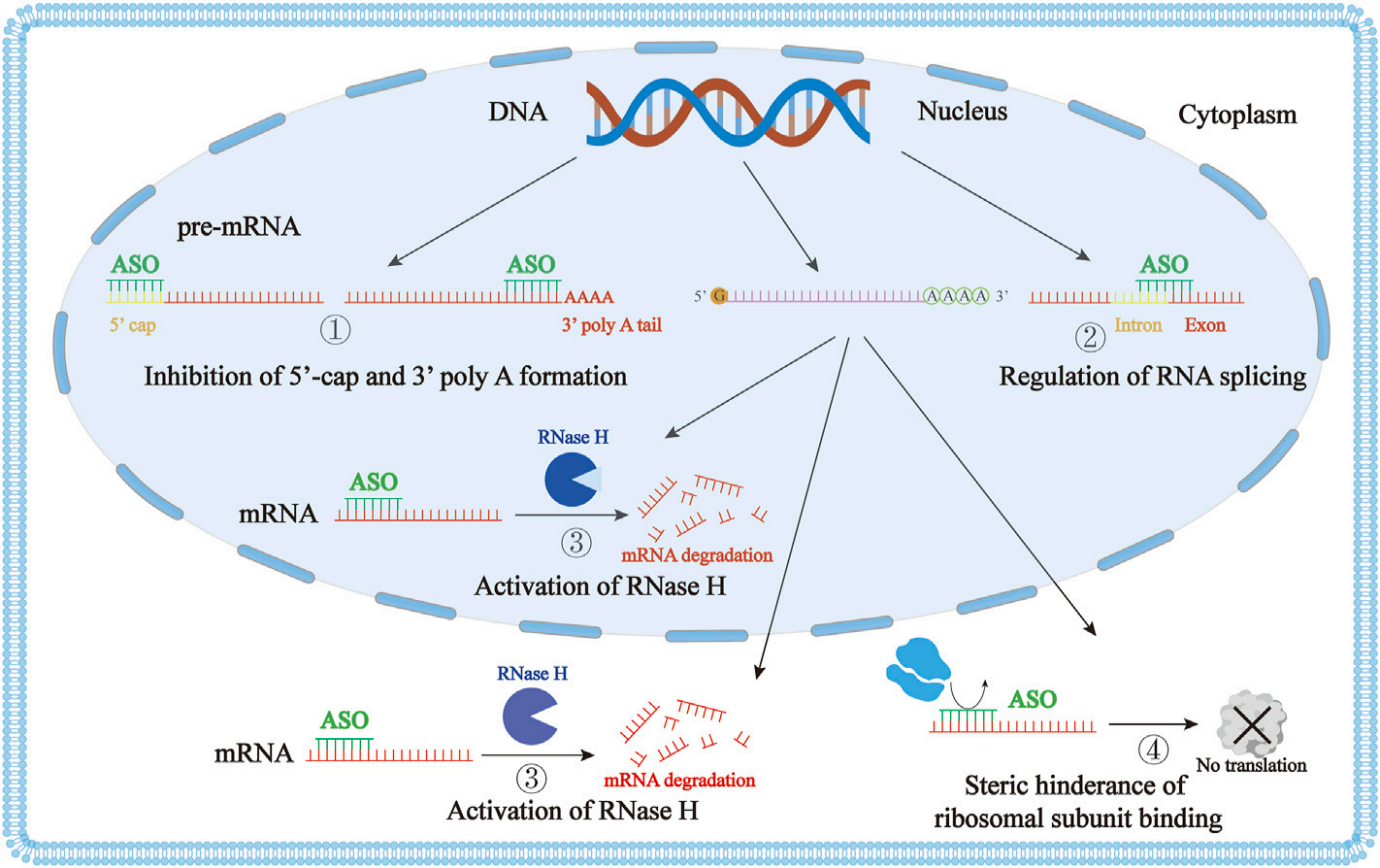
The mechanism of action of antisense oligonucleotide (ASO). ① ASO affects mRNA maturation by inhibition of the 5′ end cap formation or by blocking the polyadenylation of the 3′ end. ② ASO can selectively regulate the splicing of precursor mRNA. ③ ASO activates the RNase H enzyme to degrade DNA-mRNA duplexes through cleavage. ④ ASO can prevent the interaction of RNA and ribosomes through steric hindrance, blocking the translation process.

ASO functions mainly through four mechanisms (Figure 1). First, **ASO can affect mRNA maturation** by entering the nucleus and acting on the precursor mRNA, inhibiting the formation of the 5′ end cap or blocking the polyadenylation of the 3′ end (Gheibi-Hayat and Jamialahmadi, 2021). Second, **ASO can selectively regulate the splicing of precursor mRNA**. ASO can selectively correct the mis-splicing process through exon skipping and exon inclusion (Pagani and Baralle, 2004; Siva et al., 2014; van der Wal et al., 2017; Yang et al., 2019). Approved drugs using this mechanism include **Eteplirsen** for exon 51 skipping, **Golodirsen** and **Viltolarsen** for exon 53 skipping, and **Casimersen** for exon 45 skipping in Dystrophin mRNA. **Nusinersen** targets the precursor mRNA of the survival motor neuron 2 (SMN2), resulting in the inclusion of exon 7 (Wurster and Ludolph, 2018) (Table 1). Third, **ASO can activate the RNase H enzyme**, which specifically cleaves RNA-DNA hybrid molecules, thus inhibiting the production of proteins (Cerritelli et al., 2009; Lai et al., 2020). **Fomivirsen** targeting CMV IE2 mRNA, **Mipomersen** targeting apolipoprotein B (ApoB) mRNA, and **Inotersen** targeting transthyretin (TTR) mRNA all use this mechanism (Table 1). Finally, **ASO can prevent the interaction of RNA and ribosome through steric hindrance**, thereby blocking the translation process (Melton, 1985; Gheibi-Hayat and Jamialahmadi, 2021). There are also a few ASO drugs that act through other mechanisms. For example, **Pegaptanib** is a nucleic acid aptamer that acts by targeting the receptor of the vascular endothelial growth factor (VEGF) (Stein and Castanotto, 2017). **Defibrotide** is a natural product of DNA depolymerization, and its mechanism of action has not yet been fully elucidated (Stein and Castanotto, 2017; Alhamadani et al., 2022).

### 2.2 Design of antisense oligonucleotide

Several key issues need to be carefully considered to ensure that ASO has maximum benefit and minimum harm as a drug. These include sequence design, base modification, delivery, toxicity, pharmacodynamics, pharmacokinetics, and other factors. The sequence design of an ASO is the first and most critical step, as the ASO sequence determines its specificity. Therefore, the following factors must be considered: First, **prediction of the secondary structure of the target RNA** (Vickers et al., 2000; Andronescu et al., 2005). It can perform this task using software such as *mfold*, *sfold*, *RNAfold* or *RNAstructure* (Zuker, 2003; Ding et al., 2004; Dowell and Eddy, 2004; Mathews et al., 2004; Mathews et al., 2016). Second, we must consider the **identification of locally conserved regions** in the RNA secondary structure that can be targeted for ASO hybridization. Third, we must consider the **identification of activity-enhanced motifs** in locally conserved regions of RNA, such as CCAC, TCCC, ACTC, GCCA, CTCT, etc., which increases antisense efficiency (Matveeva et al., 2000). Also, it has shown that higher GC content in the ASO sequence produces a better effect (Ho et al., 1996). Finally, we must consider **the binding energy prediction**, where the thermodynamic binding energy between ASO and mRNA can be predicted using *RNAstructure* software. When the binding energy between ASO and mRNA is greater than or equal to −8 kcal/mol, the effect of the ASO is better (Matveeva et al., 2000). Following this basic process of ASO design can help to identify and create efficient ASO sequences. Nevertheless, experimental validation is still required to screen the overall effectiveness of the newly designed ASO.

### 2.3 Modification of antisense oligonucleotide

ASO needs to be modified to avoid degradation by nucleases in the organism. Based on the type of modification, ASO has been divided into three generations. The first generation uses a **phosphorothioate (PS) modified backbone**, that is, the non-bridge oxygen atom on the 3′-phosphate bond is replaced with a sulfur atom, which can enhance the resistance of ASO to nucleases, but reduce their affinity to the target RNA (Deleavey and Damha, 2012; Eckstein, 2014; Juliano, 2016). Among the approved ASO drugs, **Fomivirsen**, **Mipomersen**, **Nusinersen**, and **Inotersen** fall under this type of modification. Compared to unmodified ASO, PS-ASO is mainly distributed in the liver, kidney, and spleen after systemic administration (Dhuri et al., 2020). The second generation uses a **2′-alkyl modification of the ribose sugar**, which improves the affinity between ASO and target RNA and has a high resistance to nucleases as well (Kurreck et al., 2002; Prakash et al., 2008). However, this modification does not support RNase H-mediated mRNA degradation, thus reducing the effectiveness of these ASOs (Crooke, 2004). Approved ASO drugs such as **Mipomersen**, **Nusinersen**, and **Inotersen** all have the 2′-*O*-methoxyethyl (MOE) modification. To overcome these limitations, researchers have combined both generation-1 and generation-2 modifications to produce chimeric molecules, termed **gapmer**, to enhance the ASO effect. The third generation of ASO possesses a variety of **chemical modifications of the nucleotide furanose ring** (Yamamoto et al., 2011). Peptide nucleic acid (PNA), locked nucleic acid (LNA), and phosphoramidate morpholino oligomer (PMO) are the most dominant third-generation ASOs (Kurreck, 2003; Gleave and Monia, 2005). PNA is a synthetic nucleic acid mimetic in which the deoxyribose phosphate backbone is replaced by polyamide (Nielsen and PNA, 2004). LNA refers to the fact that there is a methylene bridge connecting the 2′ oxygen and 4′ carbon of ribose (Fluiter et al., 2009). PMO refers to the substitution of its pentose sugar by a morpholine ring and phosphodiester bonds by phosphoramide bonds (Summerton and Weller, 1997). The four approved PMO-modified drugs (**Eteplirsen**, **Golodirsen**, **Viltolarsen**, **Casimersen**) are all for the treatment of Duchenne muscular dystrophy (DMD). These three modifications further enhance target affinity, nuclease resistance, biostability, and pharmacokinetics (Yamamoto et al., 2011). In fact, we often use three generations of modifications in combination for better ASO effects.

The enzyme RNase H specifically cleaves RNA-DNA duplexes. For the RNA-ASO complex to be a substrate for the RNase H enzyme, at least five adjacent deoxynucleotides must be present in the ASO, and the enzyme activity is optimal when there are 8–10 adjacent nucleotides in the ASO (Crooke et al., 2021). Thus, the gapmer ASO is composed of 8–10 adjacent phosphorothioate-modified nucleotides in the middle, flanked (5′ and 3′ directions) by several 2′-alkylated modified nucleotides (Chan et al., 2006; Crooke et al., 2021). With the development of third-generation modified ASO, gapmer ASO also includes the unmodified nucleotide in the middle and the third-generation modification (such as the locking nucleotide) at both ends (Dhuri et al., 2020). While most of the above types of modification are well tolerated, some modifications have been reported to have pro-inflammatory, hepatotoxicity, nephrotoxicity, and thrombocytopenia toxicity (Frazier, 2015). For example, LNA and constrained ethyl (cEt) modifications are predisposed to hepatotoxicity (Burel et al., 2016; Kakiuchi-Kiyota et al., 2016; Kamola et al., 2017). However, as research deepens, hepatotoxicity will improve correspondingly. It has been reported that the addition of the 2′-*O*-methyl (2′-OMe) at position 2 of 3-10-3 cEt gapmer can reduce hepatotoxicity by reducing protein interactions (Shen et al., 2019). Recently, it was furthermore reported that PS substitution with mesyl-phosphoramidate (MsPA) at gap positions 2 and 3 improves RNase H cleavage, which not only does not affect ASO efficacy but also reduces hepatotoxicity (Zhang et al., 2022). It is important to note that when ASO binds to a specific ligand, it can be well delivered to a specific organ. For example, N-acetylgalactosamine (*GalNAc*) coupled to ASO allows it to achieve liver-specific delivery by binding to the asialoglycoprotein receptor (ASGPR) of hepatocytes (Crooke et al., 2019; Debacker et al., 2020).

### 2.4 Delivery of antisense oligonucleotide

ASO needs to traverse the biofilm system in order to bind to RNA and exert its effects. It is well known that biofilm systems are lipophilic, and although single-stranded ASO is amphiphilic (Crooke et al., 2018), delivery is also challenging. Cells can take up ASO through pinocytosis, adsorption and receptor-mediated endocytosis, with the specific pathways utilized being related to the chemical structure of the ASO. To address this obstacle, many delivery methods have been developed. As mentioned above, *GalNAc*-conjugated ASO can achieve targeting effect in hepatocytes, delivered by binding to the receptor (Debacker et al., 2020). The application of *GalNAc*-conjugated ASO to liver disease research is described below. In addition, many delivery systems based on polymeric nanoparticles, such as poly (lactic-co-glycolic acid) (PLGA), and polyethyleneimine (PEI), have been developed for ASO delivery (Schiffelers et al., 2004; Zhu et al., 2015). Among them, PLGA, which is biocompatible and FDA-approved, is widely used as a nanoparticle carrier for the delivery of ASOs (Danhier et al., 2012; Cai et al., 2017). Finally, several lipid-based delivery systems, lipid complexes, liposomes, and lipid nanoparticle (LNP) are also widely used to deliver ASOs (ur Rehman et al., 2013; Mahmoodi Chalbatani et al., 2019). LNPs are commonly coated with cholesterol, phospholipids, and polyethylene glycol (PEG), which can increase the stability and membrane fusion of LNP, contribute to encapsulation efficiency and endosomal escape, and minimize aggregation and immune cell opsonization (Zhang et al., 2021). In addition, extracellular vesicles are also studied and applied in ASO delivery as natural liver accumulation drug-delivery vehicles (Zhang et al., 2020).

## 3 Application of antisense oligonucleotide in liver diseases

**FIGURE 2:**
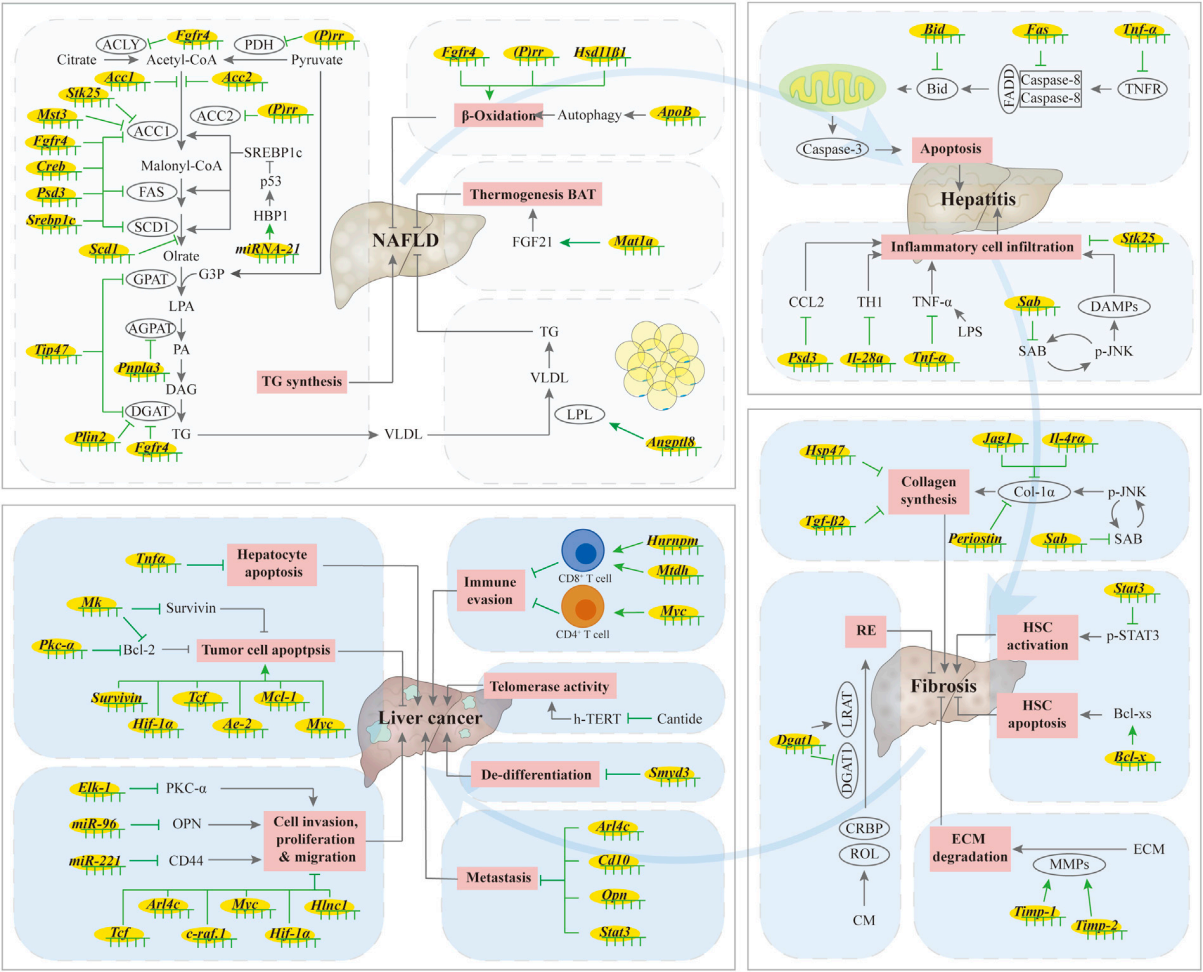
Summary of ASO in liver diseases. When the liver is under stress, including viral infection, alcohol abuse, autoimmune disease, drug use, and metabolic diseases, hepatic lipid synthesis increases, fatty acid oxidation decreases, and communication with adipose tissue is blocked, resulting in increased hepatic lipid accumulation and simple hepatic steatosis. Excessive accumulation of lipids in the liver leads to lipotoxicity, leading to ballooning degeneration of hepatocytes, infiltration of inflammatory cells, excessive secretion of inflammatory factors, and death of hepatocytes, which causes hepatitis. The activation of hepatic stellate cells (HSCs) is an important step in liver fibrosis. When hepatic fibrosis occurs, the synthesis and accumulation of extracellular matrix proteins, including collagen, increases, and the production of retinol ester (RE) by HSCs stops. The up-regulation of telomerase activity and hepatocyte apoptosis can lead to the occurrence of liver cancer, while the proliferation, migration, invasion and liver metastasis of tumor cells, as well as the de-differentiation of tumor cells, can enhance the progression of liver cancer. However, ASOs can alleviate the occurrence and development of liver diseases by regulating these processes. ↑/⊥: indicates the promoting or inhibiting effect of ASOs.

Due to the robust development of ASO technology, ASOs are being investigated as promising drugs for liver-related diseases. In the next section, we provide an overview of recent developments in the field of ASO therapeutics for liver diseases, including non-alcoholic fatty liver disease (NAFLD), hepatitis, fibrosis, and cancer. We also summarize the current preclinical trials in progress for these different stages of liver diseases (Figure 2; Table 2). The ASOs described below, except for Bcl-x, which acts by inducing alternative splicing, all act by reducing mRNA or protein levels, thereby improving the corresponding disease indicators.

### 3.1 Application of antisense oligonucleotide in non-alcoholic fatty liver disease

The current common fatty liver condition is termed NAFLD (Allison, 2004). It is caused by factors other than excessive alcohol use. NAFLD is a metabolic disorder associated with excessive accumulation of triglycerides and other lipids in the liver. It is becoming the leading cause of chronic liver disease and liver transplantation worldwide, with a prevalence as high as about 25% (Younossi et al., 2019). We consider it to be a complex disease in which the interaction between the environment and the susceptible genetic background determines the symptoms and influences the progression of the disease. The first line of treatment is lifestyle changes, especially dietary changes and exercise, but the effectiveness of this approach varies from person to person (Reinehr et al., 2009; Koot et al., 2011; Ramon-Krauel et al., 2013). With increased demand, the development of fatty liver therapy drugs is advancing rapidly, and ASO-based drugs are being strongly considered.

Several genome-wide associations and large candidate gene studies have identified the I148M patatin-like phospholipase domain protein 3 (PNPLA3) variation as the main co-genetic determinant of NAFLD (Romeo et al., 2008; Trépo et al., 2016; Eslam et al., 2018). Two studies confirmed that *Pnpla3*-ASO could alleviate hepatic steatosis in mice and rats (Kumashiro et al., 2013; Lindén et al., 2019). *Pnpla3-*ASO can decrease the level of 1-acylglycerol-3-phosphate O-acyltransferase (AGPAT) to affect the esterification of fatty acids, thereby reducing the synthesis of phosphatidic acid (PA) (Kumashiro et al., 2013). A mutation of leucine 186 to threonine in Pleckstrin and sec7 domain-containing 3 (PSD3) has been reported reducing the risk of fatty liver (Mancina et al., 2022). As predicted, *Psd3-*ASO downregulates the expression of *PSD3* in mice induced by a NASH diet, and decreases the expression of the lipogenic enzymes acetyl-CoA carboxylase (ACC), fatty acid synthase (FAS), and stearoyl-CoA desaturase 1 (SCD1), which consequently alleviates hepatic steatosis (Mancina et al., 2022). Whole genome-wide and exome studies have found that genetic variants near the γ-secretase subunit nicastrin (Ncst) are associated with HDL cholesterol (HDL-c) and LDL cholesterol (LDL-c) levels. Treatment with *Ncst*-ASO lowers triglyceride (TG) and cholesterol levels, thereby alleviating liver steatosis (Kim et al., 2018; Kim et al., 2020).

Effective regulation of lipid synthesis can alleviate hepatic steatosis. In this process, sterol regulatory element binding proteins (SREBPs) mainly regulate the expression of liver lipid synthesis genes such as *FAS*, *ACC*, and *SCD1* (Postic and Girard, 2008). Accordingly, *Srebp-1c*-ASO treatment reduced the accumulation of hepatic triglycerides and improved hepatic steatosis in mice with NAFLD (Frederico et al., 2011). Diacylglycerol acyltransferase (DGAT) is an enzyme required for the last step of triglyceride synthesis and it includes two isoforms: DGAT1 and DGAT2. Studies have found that *Dgat2*-ASO affects the synthesis of liver fat and alleviates liver fat in obese mice (Yu et al., 2005; Choi et al., 2007). In addition, DGAT2 also has its corresponding antisense drug IONIS-DGAT2_RX_ (ClinicalTrials.gov NCT03334214), which is currently in phase 2 of clinical trials for the treatment of NASH (Loomba et al., 2020). The cAMP-response element binding protein (CREB) is a key transcription factor in regulating the expression of multiple gluconeogenesis genes, and its expression in the liver has a regulatory effect on liver lipid production. *Creb*-ASO improves liver fat degeneration in type 2 diabetic rats by inhibiting the expression of lipid synthesis related genes (Erion et al., 2009).

The accumulation of lipid droplets (LDs) increases in the fatty liver, and some LD proteins, such as perilipin 2 (PLIN2, also called ADRP) and perilipin 3 (PLIN3, also called TIP47), regulate LDs enlargement (Brasaemle, 2007; Buers et al., 2009). *Adrp/Plin2-*ASO improves diet-induced insulin resistance and inhibits the expression of lipid synthesis-related genes, thereby decreasing liver fat accumulation (Varela et al., 2008; Imai et al., 2012). Similarly, *Tip47/Plin3*-ASO affects liver LDs and improves liver steatosis (Carr et al., 2012). Overexpression of serine/threonine protein kinase (STK) 24, also known as MST3, increases the number and size of LDs in the liver (Cansby et al., 2019a). Its mRNA expression level was also positively correlated with liver-related disease. Targeting MST3 using *Mst3*-ASO reduces the levels of *ACC in vivo*, thus reducing liver lipids and slowing the progression of diet-induced NAFLD (Caputo et al., 2021). Similarly, *Stk25*-ASO modified by *GalNAc* can ameliorate steatosis by regulating the expression of lipid synthesis genes, and reducing the infiltration of macrophages to improve hepatic steatohepatitis (Nuñez-Durán et al., 2018; Cansby et al., 2019b).

MicroRNAs (miRNAs) are small non-coding RNAs that regulate gene expression at the post-transcriptional level by inhibiting mRNA translation or promoting mRNA degradation (Correia de Sousa et al., 2019). A total of 23 miRNAs were observed to be underexpressed or overexpressed in NASH (Cheung et al., 2008). Treatment with *miRNA-21*-ASO improves steatosis by increasing the expression of its target gene *HBP1*, a transcriptional activator of p53, which then blocks downstream lipid biosynthetic genes (Wu et al., 2016a).

In addition to reducing lipid synthesis, increasing fatty acid oxidation also alleviates fatty liver. *Acc-*ASO, fibroblastic growth factor receptor 4 (*Fgfr4*)-ASO and prorenin receptor (*[P]rr*)-ASO can ameliorate hepatic steatosis by regulating fatty acid oxidation and lipid synthesis (Savage et al., 2006; Yu et al., 2013; Ren et al., 2018). In addition, hydroxysteroid 11-beta dehydrogenase 1 (*Hsd11β1*)-ASO decreases *de novo* lipid synthesis while increasing fatty acid oxidation (Li et al., 2011a). Treatment with *Scd1*-ASO not only reduces the expression of genes related to lipid synthesis (*FAS*, *ACC1*, *Srebp-1*) but also increases the expression of genes (such as carnitine palmitoyltransferase 1, *cpt-1*) related to fatty acid oxidation (Jiang et al., 2005). Treatment with *ApoB*-ASO increases endoplasmic reticulum stress and autophagy, resulting in increased fatty acid oxidation (Conlon et al., 2016). In sum, all three treatments ameliorated hepatic steatosis.

The intercommunication between adipose tissue and liver tissue is critical for maintaining proper physiological homeostasis. In mice, treatment with *Angptl8*-ASO increases the amount of lipid absorption and prevents the ectopic accumulation of fat in the liver, thus improving liver steatosis (Vatner et al., 2018). Application of *Mat1a*-ASO promotes brown adipose tissue (BAT) thermogenesis and improves fatty liver in both *ob/ob* and high-fat diet (HFD) induced mice (Sáenz de Urturi et al., 2022). Fatty acid binding protein (FABP) 3 has been shown to be involved in HFD-induced liver steatosis in zebrafish, and *Fabp3*-ASO reduces liver lipid accumulation (Shimada et al., 2015). Taken all together, ASOs can prevent fatty liver by regulating hepatic lipid metabolism, the crosstalk between adipose tissue and the liver, and other processes (Figure 2).

### 3.2 Application of antisense oligonucleotide in hepatitis

NASH affects the health and quality of life for 3%–5% of the global population (Povsic et al., 2019). Inflammation and apoptosis both accompany hepatitis, alcoholic liver disease, liver fibrosis, and cirrhosis. The most severe forms of inflammation result in extensive hepatocyte death, severe liver damage, and ultimately death, with a severely elevated mortality rate due to the lack of specific treatment. Fortunately, the use of ASO as an alternative therapy has been reported as a viable treatment for hepatitis.

In most cases, inflammation is caused by the activation of T cells and macrophages, which directly affect hepatocytes or secrete pro-inflammatory factors such as interleukins (IL-1, IL-6, IL-12) and tumor necrosis factors (TNF-α) (Martich et al., 1991; Vergani et al., 2002). Tumor necrosis factor receptor-related proteins (Fas and TNFR1, etc.) play an essential role in this process. TNF-α-induced apoptosis is an early result of liver failure and liver injury (Leist et al., 1995; Kawaguchi et al., 1999). Treatment with *Tnf-α-*ASO (TJU-2755), which is delivered into kupffer cells by galactosylated low molecular weight chitosan, alleviates the symptoms of severe hepatitis in mice (Dong et al., 2009). Moreover, treatment with *Tnf-α*-ASO also improves lipopolysaccharide (LPS)-induced hepatitis (Mochizuki and Sakurai, 2011). Fas, a TNF receptor family protein, also plays a role in abnormal apoptosis, and treatment with modified-*Fas*-ASO (Ionis 22023) protects mice from severe hepatitis (Zhang et al., 2000). Among the apoptosis proteins, the Bcl-2 protein family, members of which either inhibit (such as Bcl-2, Bcl-xL, Bcl-w, A1, Mcl-1, and Boo, etc.) or promote (such as Bax, Bak, Bok, Bik, Bad, and Bid, etc.) apoptosis, has been adequately studied. Accordingly, *Bcl-xL*-ASO (Ionis 16009) increases Fas-induced hepatocyte apoptosis and increases the lethality of severe hepatitis in mice. Conversely, *Bid-*ASO (Ionis 119935) protects mice from death and also protects mice from Fas-induced hepatocyte apoptosis when both genes are silenced simultaneously (Zhang et al., 2003). T cell-mediated explosive hepatitis is a potentially life-threatening condition. IL-28A (IFN-λ2) plays a regulatory role in explosive hepatitis, and *Il-28a*-ASO can also reduce T cell-mediated hepatitis in Con A-induced hepatitis (Siebler et al., 2007).

Treatment with *Jnk1*-ASO can improve hepatitis induced by HFD (Singh et al., 2009). Mitochondrial SH3 homology associated BTK binding protein (SH3BP5, also named SAB), as a JNK target, plays a role in high-fat, high-calorie, high-fructose (HFHC) diet induced NASH. Treatment with *Sab-*ASO modified by *GalNAc* reduces inflammatory cell infiltration into the liver, alleviates NASH-related symptoms, reduces collagen deposition and diminishes liver fibrosis-related symptoms (Win et al., 2021). Treatment with *Psd3*-ASO reduces the hepatic lobule inflammation score, and has a certain remission effect on steatohepatitis in a mice model for NASH (Mancina et al., 2022). STK25 also has a regulatory role in hepatic steatohepatitis. *Stk25-*ASO reduces macrophage infiltration and improves the condition of hepatitis to a certain extent (Cansby et al., 2019b). Taken altogether, the use of ASOs can ameliorate severe hepatitis by affecting the apoptosis of hepatocytes in steatohepatitis (Figure 2).

### 3.3 Application of antisense oligonucleotide in liver fibrosis

Liver fibrosis is a wound healing response characterized by excessive deposition of extracellular matrix proteins, including collagen, in the liver. The activation of hepatic stellate cells (HSCs) is a marker of liver fibrosis. Fibrosis is an advanced stage in the progression of various chronic liver diseases to cirrhosis and cancer. When effectively treated at the fibrosis stage, chronic liver disease can still be prevented from becoming a more severe instance. Therefore, it is particularly critical to develop therapeutic ASO drugs for this phase.

Myriad cellular regulators contribute to the process of liver fibrosis. Matrix metalloproteinases (MMPs) are the main components of hydrolyzing the extracellular matrix. Their activities are regulated by matrix metalloproteinase inhibitors (TIMPs). The role of TIMPs in liver fibrosis has long been studied and TIMP-1 and TIMP-2 have been identified as being the most prominent. Treatment with *Timp-1* and *Timp-2*-ASO reduce their mRNA levels as well as collagen accumulation, and consequently improve liver pathology and liver function (Nie et al., 2001; Nie et al., 2010). Heat shock protein 47 (HSP47) is a specific molecular chaperone necessary for collagen synthesis, and its expression increases in parallel with the mRNA level of procollagen during liver fibrogenesis (Masuda et al., 1994). Based on this observation, Brown et al. (2005) used *Hsp47*-ASO to inhibit HSP47 expression, and found that this could reduce procollagen synthesis. This indicates that *Hsp47*-ASO can be used as an anti-fibrotic treatment (Brown et al., 2005). TGF-β2 is a member of the TGF-β family and plays a role in liver fibrosis, and *Tgf-β2*-ASO decreases collagen deposition to alleviate liver fibrosis (Dropmann et al., 2020).

Aberrant Notch activity in hepatocytes has been shown to be crucial for the development of NASH-induced liver fibrosis, and increased expression of the Notch ligand Jag1 has been closely associated with Notch activation in human liver biopsies and in mouse NASH models. In mice, treatment with *Jag1*-ASO alleviates NASH diet-induced liver fibrosis (Yu et al., 2021). IL-4Rα is activated by the regulation of IL-4 and IL-13, and is the central switch in the transition of M1 macrophages into the M2 type. IL-4Rα activation regulates the functional polarization of macrophages and promotes the development of fibrosis. Treatment with *Il-4rα-*ASO is effective in attenuating liver fibrosis (Weng et al., 2018).

Targeting HSCs is a modern perspective regarding the treatment of liver fibrosis. Periostin is a matrix-secreting protein with multiple roles in tissue development and regeneration. It activates HSCs by interacting with the α_v_ integrin, and reduces hepatic fibrosis in hepatotoxicity and cholestatic liver injury when it is absent (Lu et al., 2014; Sugiyama et al., 2016). Treatment with *Periostin-*ASO reduces liver fibrosis in mice, which is reflected in the reduction of the alpha-smooth muscle actin (α-SMA), collagen type I, and other fibrotic markers (Kobayashi et al., 2020). The signal transducer, activator of transcription 3 (STAT3), drives the activation of HSCs (Xiang et al., 2018). Targeting STAT3 in HSCs by using exosomes to carry the *Stat3*-ASO significantly reduces the level of STAT3 mRNA, thereby inhibiting the activation of HSCs and thus improving liver fibrosis (Tang et al., 2021). It has been shown that the apoptosis of HSCs is related to the regression of hepatic fibrosis (De Minicis et al., 2012). Remarkably, treatment with a splicing ASO against Bcl-x transforms anti-apoptotic Bcl-x_L_ to pro-apoptotic Bcl-x_S_, thus inducing apoptosis of HSCs and improving liver fibrosis (Wu et al., 2016b).

The production of retinol esters (RE), which is catalyzed by lecithin-retinol acyltransferase (LRAT) and acyl-CoA retinol acyltransferase (ARAT), disappears in HSC activation and liver fibrosis. DGAT1, an enzyme that catalyzes the last step of triglyceride biosynthesis, exhibits additional ARAT activity *in vitro*. Treatment with *Dgat1-*ASO inhibits the activation of HSCs in mice and rats, increases the expression of LRAT, promotes the accumulation of RE in the liver, and alleviates liver fibrosis (Yamaguchi et al., 2008).

Taken together, ASOs can ameliorate liver fibrosis by reducing the synthesis of ECM protein components, increasing their degradation, affecting HSCs activation, regulating lipid synthesis in HSCs, as well as by regulating inflammation (Figure 2).

### 3.4 Application of antisense oligonucleotide in liver cancer

Liver cancer is a global health challenge with increasing incidence worldwide (Villanueva, 2019). It is estimated that by 2025, one million people will develop liver cancer each year. Liver cancer mainly consists of primary liver cancer and secondary liver cancer. Primary liver cancers include hepatocellular carcinoma (HCC) and intrahepatic cholangiocarcinoma, as well as other fewer common types. Among various cancers, HCC is the most common aggressive malignancy in the world. Regarding the treatment of liver cancer, there are currently no interventions to prevent the development of HCC in high-risk patients, and the effectiveness of different treatment options may be limited due to genetic variability among patients. Therefore, it is necessary to find a personalized treatment plan, and ASO drugs provide excellent potential options.

The application of ASO in liver cancer has been explored for a long time. STAT3 is a convergence point of numerous oncogenic signals. Treatment with *Stat3-*ASO reduces the circulating vascular endothelial growth factor, basic fibroblast growth factor, tumor volume and weight in HCC, and also prolongs survival in mice (Li et al., 2006). In the process of cancer treatment, radiotherapy is a commonly used approach. However, during radiotherapy, radiation usually causes the apoptosis of hepatocytes and subsequent liver damage, which limits the efficacy of this treatment (Terashima et al., 1995). TNFR1 is an influential factor in the apoptosis signaling pathway. Treatment with *Tnfr1*-ASO before radiation reduced radiation-induced apoptosis in hepatocytes as well as micronucleus formation (Huang et al., 2006).

Telomerase is a unique ribonucleoprotein complex that plays an important role in cell immortality and tumorigenesis (Holt and Shay, 1999). Studies have shown that telomerase is activated in human tumors, but not in adjacent normal cells (Shay and Bacchetti, 1997), suggesting that telomerase is a target for cancer diagnosis and treatment. Human telomerase reverse transcriptase (h-TERT) is the catalytic subunit of a telomerase. Studies have found that 90% of examined tumors show a relation between telomerase activity and the expression of the activity-limiting component h-TERT (Counter et al., 1998; Yang et al., 2002). Cantide, the *h-TERT* antisense nucleic acid drug, inhibits tumor cell growth and reduces h-TERT mRNA expression and telomerase activity (Du et al., 2003). Cantide also significantly inhibits the growth of *in situ* tumors and prevents intrahepatic tumor recurrence and lung metastasis (Lin et al., 2005). In addition, treatment with *Tert-*ASO also inhibits the invasion and tumor progression of liver cancer caused by TERT activation (Ningarhari et al., 2021). Promoting apoptosis of tumor cells is an important strategy in the treatment of liver cancer. PKC-α is highly expressed in rat hepatoma cells, and treatment with *Pkc-α-*ASO reduces the expression of anti-apoptotic protein Bcl-2, promotes cell apoptosis, and inhibits the growth of tumor cells (Lin et al., 2000). Mcl-1 and survivin are members of the class of anti-apoptotic proteins associated with cell apoptosis and the cell cycle, and are highly expressed in malignant cells. Treatment with *Mcl-1-*ASO and *Survivin*-ASO (LY218308) promote hepatoma carcinoma cell apoptosis (Sieghart et al., 2006; He et al., 2007; Carrasco et al., 2011; Dai et al., 2012; Wang et al., 2021). Heparin binding factor midkine (MK) is also highly expressed in a variety of malignant tumors and treatment with *Mk*-ASO inhibits the proliferation of tumor cells, reduced the expression of anti-apoptotic proteins survivin and Bcl-2, and promoted apoptosis in tumor cells (Dai et al., 2006; Dai et al., 2007; Dai et al., 2009). Furthermore, antisense therapy using *Hif-1α* (hypoxia-inducible factor-1), or *Ae-2* (anion exchanger 2) has also been found to induce apoptosis (WeiXing et al., 2008; Hwang et al., 2009).

Inhibiting the migration and invasion of tumor cells is critical to stopping the spread of cancer. The ETs protein family of transcription factors regulates the expression of oncogenes and tumor suppressor genes, and plays an important role in the proliferation, survival, and differentiation of normal cells (Oikawa, 2004). ETs-like transcription factor 1 (Elk-1) is a nuclear transcriptional co-activator regulating the expression of ETs protein family genes, and the activation of Elk-1 is necessary for tumor progression (Treisman, 1992; Price et al., 1995; Xiao et al., 2002). Inhibiting the expression of Elk-1 reduces the expression of PKC-α, as well as tumor migration and invasion in HCC (Hsieh et al., 2006). Transfection of *Elk-1-*ASO reduces cell proliferation and inhibits tumor growth in mice (Ying et al., 2008).

CD44 is one of the essential tumor-initiation cell markers in HCC (Yang et al., 2008; Zhu et al., 2010) and it is involved in regulating the invasion and migration of HCC cells. miR-221 regulates the expression of CD44 in HCC, and treatment with *miR-221*-ASO downregulates CD44 protein expression (Kim et al., 2017). Osteopontin (OPN) is associated with liver cancer metastasis and invasion, and treatment with *Opn*-ASO inhibits liver cancer metastasis (Chen et al., 2011). In addition, treatment with *miR-96-*ASO decreases the expression of OPN, thus preventing the invasion of liver tumors (Chen et al., 2012). MYC promotes tumorigenesis by inducing proliferation, blocking differentiation, and promoting self-renewal (Dang, 2012; Gabay et al., 2014). Thus, treatment with *Myc-*ASO reduces cell proliferation and induces apoptosis, thus reducing tumor growth and invasion (Li et al., 2011b; Yuan et al., 2014; Dhanasekaran et al., 2020). ADP-ribosylation factor-like 4C (ARL4C) is a member of the small GTP-binding protein family and plays a role in tumor genesis and development. Treatment with *Arl4c*-ASO inhibits the proliferation and metastasis of primary and metastatic liver tumors (Harada et al., 2019). Long non-coding RNA-HLNC1 antisense therapy inhibits tumor growth (Qian et al., 2020), and *HIF-1α*, *c-raf.1* antisense therapy inhibits cell proliferation (WeiXing et al., 2008; Das et al., 2010).

Immune escape also plays an essential role in the development of tumors. The heterogeneous nuclear ribonucleoprotein M (HNRNPM) is involved in tumor immune escape and is elevated in liver cancer. Treatment with *Hnrnpm*-ASO promotes activation of CD8^+^ T cells, thus blocking immune escape and improving tumor treatment (Zhu et al., 2022). Metadherin (MTDH) is expressed in a variety of malignancies (Hu et al., 2009; Yang et al., 2017), and treatment with *Mtdh-*ASO increases CD8^+^ T cells and enhances tumor immunity (Wan et al., 2022). MYC oncogenes also promote tumor formation by inducing immune escape, and treatment with *Myc-*ASO increases the number of CD4^+^ T cells and reshapes the tumor microenvironment (Dhanasekaran et al., 2020).

Cell dedifferentiation is also a hallmark of liver cancer. Dedifferentiation processes in cancer are pivotal for the generation and expansion of cancer stem cells, which play a role in cancer cell renewal and metastatic potential, and correlate with the level of aggressiveness and drug resistance of the tumor (Nikolaou et al., 2015; Atashzar et al., 2020; Schulte et al., 2020). The oncogenic function of suppressor of variegation, enhancer of zeste, and Myeloid-Nervy-DEAF1-domain family methyltransferase Smyd3 has been implicated in various malignancies, including HCC. Treatment with *Smyd3-*ASO blocks the cell dedifferentiation process and reduces the burden of DEN-induced liver cancer (Kontaki et al., 2021).

Secondary liver cancer is due to liver metastasis caused by other cancers, a process in which tumor cells from other organs migrate to the body and colonize new locations, including adenocarcinoma liver metastasis, colorectal liver metastasis, etc. The expression of CD10 has been correlated with liver metastasis of colorectal cancer (Fujimoto et al., 2005; Fujita et al., 2007) and treatment with *Cd10*-ASO inhibits CD10 expression and reduces the number and size of liver metastases in nude mice (Luo et al., 2009).

In summary, ASO can regulate the proliferation, migration, and invasion of tumor cells, as well as regulate immunity and liver metastasis by affecting multiple genes and proteins, and may also improve therapeutic effects of radiotherapy by mitigating side effects during the treatment process (Figure 2).

## 4 Challenges and perspectives of antisense oligonucleotide in liver diseases

Liver diseases are rapidly becoming widespread, and their treatment is a clinical challenge. The use of ASO, as a potential therapeutic for liver diseases, has attracted great attention in recent years. ASO has superior tissue distribution in the liver after intraperitoneal and subcutaneous administration (Geary et al., 2015). The use of *GalNAc-*coupled ASO enables the targeting of ASO in the liver, which is also a major advance in the field of ASO delivery. Thus, the concentration and persistence of ASO in the liver after treatment produces a high tissue distribution, giving ASO potential applications in both the early and late stages of liver-related diseases.

Despite these positive outlooks, the withdrawal of Fomivirsen and Mipomersen from the market indicates the potential for drug toxicity and side effects in ASO drugs. Therefore, several issues must be kept in mind when developing ASO drugs for liver diseases. First, ASO-mediated hepatotoxicity is a primary challenge. Studies have shown that locked nucleic acid modification can enhance the biological effect of the ASO, but can also bring about significant hepatotoxicity (Swayze et al., 2007; Burdick et al., 2014; Kasuya et al., 2016). In addition, GAP-modified ASO (Gapmer) has also been found to cause severe hepatotoxicity due to the RNA off-target cleavage effect (Kasuya and Kugimiya, 2018). Second, nephrotoxicity was also found in the use of ASO (Geary et al., 2015; Moisan et al., 2017). Fortunately, *GalNAc-*coupled ASO shows reduced nephrotoxicity (Sewing et al., 2019). In addition, the double coupling of cholesterol and *GalNAc* reduced the distribution of ASO in the kidney, which also lowered nephrotoxicity (Wada et al., 2018). The third issue we need to be mindful of is that ASO is readily degraded in plasma and is prone to off-target effects (Kulkarni et al., 2021). By constructing liver-specific or whole-body monoacylglycerol acyltransferase 1 (*Mogat1*) knockout mice, Lutkewitte et al. (2021) found that *Mogat1* knockout did not affect glucose tolerance, but *Mogat1-ASO* treatment could improve glucose tolerance in wild-type and *Mogat1* knockout mice, suggesting that *Mogat1-*ASO treatment had an off-target effect. Meanwhile, hybridization-dependent toxicity also mediates off-target effects in splice-switching ASOs (Scharner et al., 2020). When the ASO binds to the RNA, RNase H recognizes the presence of the DNA-RNA duplex and then degrades the RNA strand. The cleavage requires merely five continuous base pairs (Wu et al., 1999), which can result in the knock-down of partially hybridized targets. The presence of locked nucleic acid and restricted ethyl nucleotide modifications in Gapmers is also prone to an off-target effect (Kamola et al., 2015; Hagedorn et al., 2017). Luckily, this effect can be reduced by extending the length of Gapmers (Yasuhara et al., 2022). Although spatial blocking oligonucleotides have the potential to bind to nearby complementary sites in the transcriptome, leading to off-target effects (Holgersen et al., 2021), this property can be avoided by RNA-Seq experimental screening (Holgersen et al., 2021). Moreover, off-target effects can be reduced during the design of ASOs. As genome research expands and continues to grow, as bioinformatics continues to improve and information technology platforms continue to evolve, target genes can be more accurately modeled in the preliminary design process for ASOs. With the aid of such information, unwanted toxic reactions due to off-target effects can be minimized.

Finally, Defibrotide was the first ASO drug approved for the treatment of VOD (Choy, 2016). There are also several ongoing clinical trials of antisense oligonucleotides for liver disease. One such is bepirovirsen, a second-generation 2′-O-methoxyethyl (MOE)-modified antisense phosphorothioate oligonucleotide (ClinicalTrials.gov NCT02981602) (Yuen et al., 2021), another is GSK3389404, which is formed by covalent binding of bepirovirsen to three GalNAcs (ClinicalTrials.gov NCT03020745) (Yuen et al., 2022), and finally, RO7062931, a GalNAc conjugated single stranded locked nucleic acid oligonucleotide (ClinicalTrials.gov NCT03038113) (Gane et al., 2021) for the treatment of HBV. In addition, the aforementioned DGAT2 and its corresponding antisense drug IONIS-DGAT2_RX_ (ClinicalTrials.gov NCT03334214) are currently undergoing phase 2 clinical trials for the treatment of NASH (Loomba et al., 2020). While all relevant applications of ASOs to liver disease reviewed in this paper are from animal model systems, various human cell lines have been used to investigate the application of human ASOs in these animal models. An example is the human-derived cells used in the ectopic naked mouse model in Section 3.4 to study liver cancer. In summary, the development of ASO drugs for the treatment of liver diseases has great potential. There is an urgent need to move ASOs from the animal level into clinical trials.

## 5 Summary

Liver-related diseases are a global epidemic and one of the leading causes of death. The discovery and development of drugs for liver-related diseases is extremely urgent. ASO, as a precise targeted drug, has great potential for the treatment of liver-related diseases.

## Glossary

ASO: antisense oligonucleotide
ARL4C: ADP-ribosylation factor-like 4c
ARAT: acyl-CoA retinol acyltransferase
ApoB: apolipoprotein B
ANGPTL8: Angiopoietin-like 8
AMD: age-related macular degeneration
AGPAT: 1-acylglycerol-3-phosphate O-acyltransferase
AE-2: anion exchanger 2
ADRP: adipose differentiation-related protein
ACC: acetyl-CoA carboxylase
ASGPR: asialoglycoprotein receptor
BAT: brown adipose tissue
CREB: cAMP-response element binding protein
CMV: cytomegalovirus
DMD: Duchenne muscular dystrophy
DGAT: diacylglycerol acyltransferase
FABP: fatty acid binding protein
Elk-1: ETs-like transcription factor 1
FDA: U.S. Food and Drug Administration
FAS: fatty acid synthase
GalNAc: N-acetylgalactosamine
h-TERT: human telomerase reverse transcriptase
HSP47: heat shock protein 47
HSC: hepatic stellate cells
HNRNPM: heterogeneous nuclear ribonucleoprotein M
HFD: high-fat diet
HDL-c: high-density lipoprotein cholesterol
HCV: hepatitis C
HCC: hepatocellular carcinoma
HBV: hepatitis B
IL-4Rα: interleukin 4 receptor A subunit
Ie2: intermediate early region 2
Jag1: jagged-1
LPS: lipopolysaccharide
LPL: lipoprotein lipase
LDs: lipid droplets
LDL-c: low-density lipoprotein cholesterol
LNA: locked nucleic acid
LNP: lipid nanoparticle
LRAT: lecithin-retinol acyltransferase
MUFAs: monounsaturated fatty acids
MTDH: metastasis adhesion protein
MST3: serine/threonine protein kinase 24
MMPs: matrix metalloproteinases
MK: midkine
Mat1a: methionine adenosine transferase 1A
MOE: 2′-O-methoxyethyl
NCST: nicastrin
OPN: osteopontin
PSD3: pleckstrin and sec7 domain-containing 3
PNPLA3: patatin-like phospholipase domain-containing 3
PKCε: protein kinase C epsilon
PDH: pyruvate dehydrogenase
PBC: primary biliary cholangitis
PA: phospholipid acid
(P)RR: Prorenin receptor
PS: phosphorothioate
PNA: Peptide nucleic acid
PMO: phosphoramidate morpholino oligomer
PEG: polyethylene glycol
RE: retinol esters
STK25: serine/threonine protein kinase 25
STAT3: signal transducer and activator of transcription 3
SREBP: sterol regulatory element binding protein
SMA: spinal muscular atrophy
SCD1: stearyl CoA desaturase 1
SAB: SH3 homology associated BTK binding protein
SMN2: survival of motor neuron 2
TNF-α: tumor necrosis factor-α
TNFR1: tumor necrosis factor receptor 1
TIP47: tail interacting protein 47
TIMPs: matrix metalloproteinase inhibitors
TG: triglyceride
TTR: transthyretin
VOD: veno-occlusive disease
VLDL: very low density lipoprotein
VEGF: vascular endothelial growth factor
